# Screening and Improvement of an Anti-VEGF DNA Aptamer

**DOI:** 10.3390/molecules15010215

**Published:** 2010-01-07

**Authors:** Yoshihiko Nonaka, Koji Sode, Kazunori Ikebukuro

**Affiliations:** Department of Biotechnology & Life science, Tokyo University of Agriculture and Technology, 2-24-21 Naka Cho, Koganei, Tokyo, 1848588, Japan

**Keywords:** aptamer, cancer diagnosis, sensor element, VEGF_121_, VEGF_165_

## Abstract

To obtain an aptamer with a high affinity for vascular endothelial growth factor (VEGF), we focused on the receptor-binding domain (RBD) of VEGF as a target epitope. Three rounds of screening gave Vap7, which bound to the VEGF isoforms VEGF_121_ and VEGF_165_ with *K*_D_ values of 1.0 nM and 20 nM, respectively. Moreover, Vap7 showed specificity within the VEGF family. Secondary structure predictions and circular dicrhoism suggested that Vap7 folds into a G-quadruplex structure. We obtained a mutant aptamer that contains only this region of the aptamer sequence. This truncated mutant (V7t1) bound to both VEGF_121_ and VEGF_165_ with *K*_D_ values of 1.1 nM and 1.4 nM, respectively. Its sequence was 5'-TGTGGGGGTGGACGGGCCGGGTAGA-3', and it appeared to form a G-quadruplex structure. We also produced an aptamer heterodimer consisting of our previously derived aptamer (del5-1), which binds to the heparin-binding domain of VEGF, linked to V7t1. The resulting heterodimer bound strongly to VEGF_165_ with a *K*_D_ value of 4.7 × 10^2^ pM.

## 1. Introduction

Aptamers are nucleic acids that bind to target molecules [1,2]. Recently, aptamers have drawn a great deal of attention as sensing elements in biosensors for small molecules and proteins [3,4,5]. DNA aptamers can be readily synthesized, chemically modified, and refolded, and they can be integrated into microarrays, microfluidics, sandwich assays and electrochemical biosensors. These advantages of DNA aptamers make them good sensing elements for the target molecule recognition. DNA aptamers with a high affinity and specificity for the marker proteins of tumors would be useful in the sensitive and accurate diagnosis of a number of diseases. Moreover, aptamers that can inhibit the activities of their target molecules are useful tools for molecular control [6,7,8]. RNA aptamers have already been used to develop a drug (Macugen^®^) against age-related macular degeneration [9].

With the aim of creating a more effective tool for the diagnosis of cancer, we considered vascular endothelial growth factor A (VEGF-A) as a target marker protein. VEGF-A is a crucial regulator of angiogenesis, which plays an important role in many diseases. Furthermore, VEGF-A stimulates the vascularization of solid tumors, and the serum level of VEGF-A is therefore a useful indicator of the presence of a tumor. It is known that VEGF-A has several isoforms that contain various numbers of residues as a result of selective splicing. VEGF_165_ is the most abundantly expressed isoform and one that plays a vital role in angiogenesis; it has two domains: a receptor-binding domain (RBD) and a heparin-binding domain (HBD). Several researchers have reported aptamers that bind to VEGF_165_ [10,11].

We have previously reported the isolation of two DNA aptamers that bind to the HBD of VEGF_165_ with *K*_D_ values of 1.3 × 10^2^ nM and 5.0 × 10^2^ nM, respectively [12]. We have also developed novel DNA aptameric sensors, one of which is based on a detection system with easy Bound/Free separation, “capturable aptamer” [13]. Another aptamer-based sensor, the aptameric enzyme subunit, has been designed to permit easy homogeneous detection [14]. 

To achieve a high sensitivity for the target molecule, it is important to use molecular recognition elements with a high affinity. We have previously reported that to improve the affinity of aptamers for their targets by means of dimerization, it is necessary to connect a pair of aptamers through a linker. This design is based on the avidity of the antibody. We previously linked two identical HBD-binding aptamers [12] through chains of thymine bases of various lengths [15]. By this means, we improved the *K*_D_ value of the aptamer against VEGF_165_ from 3.0 × 10^2^ nM to 17 nM. 

An aptamer that binds to the RBD rather than the HBD of VEGF would be useful for the construction of an aptamer heterodimer containing the previously obtained HBD-binding aptamer. An aptamer heterodimer consisting of both an HBD-binding aptamer and an RBD-binding aptamer would be expected to have a lower *K*_D_ value than those of the individual aptamer components. Moreover, two aptamers that bind to different regions of the target protein should permit the construction of a sandwich assay system. With this idea in mind, we attempted to obtain a new RBD-binding aptamer. To achieve this objective, we carried out aptamer screening with VEGF_121_, an isoform of VEGF-A that the RBD but not the HBD. In this manner, we tried to select and improve specific DNA aptamers against VEGF that could be incorporated into sensor elements for cancer diagnosis.

## 2. Results and Discussion

### 2.1. Screening of the Aptamers

Aptamers against the VEGF family were isolated by using the systematic evolution of ligands by exponential enrichment (SELEX) method. In the selection process, VEGF_121_ was immobilized on a membrane that was then incubated with fluorescein isothiocyanate (FITC)-labeled oligonucleotides. The binding affinities of the oligonucleotides in a single-strand DNA (ssDNA) library for VEGF_121_ were evaluated at each round by an aptamer blotting assay. After three rounds of selection, eight clones that appeared to bind to VEGF_121_ were analyzed (Table 1); there were no overlapping sequences.

**Table 1:** Sequence obtained after three rounds of screening. The table shows only the random sequence region. All aptamers had a Fw primer sequence at the 5'-end and a complimentary Rev primer sequence at the 3'-end. (The sequences of the primer are given in the Methods section in the SELEX protocol subsection).

Name	Sequence (5' to 3')	Length
Vap1	CGCTAGGGGGTGGAGGGCTTCGAGGGGACT	30 mer
Vap2	GTCTCTGTGACTCTTGTGGGGGCCGCGTCA	30 mer
Vap3	AGTTCGTCCGAGGTTCTGTGTTTGGTGCC	29 mer
Vap4	CATTGCAACATACTATCTGGTGGGCCAAAG	30 mer
Vap5	CGCCTTGCATGGTACGGGGTCTCGACGAGC	30 mer
Vap6	GGATGTGTCTTGCGAGATCACCACCGGCCC	30 mer
Vap7	GCACTCTGTGGGGGTGGACGGGCCGGGT	28 mer
Vap8	CAAGATCACTGGTTGCGCGCGTGTGCCCCCC	31 mer

**Figure 1:**
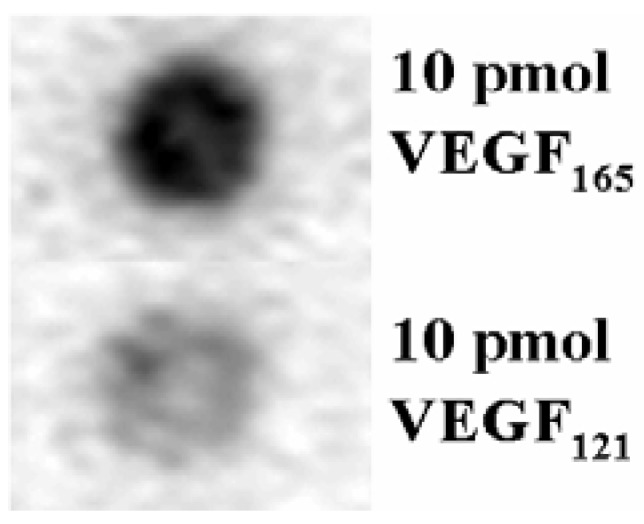
Evaluation of aptamer binding against the VEGF family. Ten pmol of each of VEGF_121_ and VEGF_165_ were immobilized on a nitrocellulose membrane. The membrane was incubated sequentially with 100nM of FITC-modified Vap7 and with HRP-labeled anti-FITC antibody. Finally, the chemiluminescence generated by HRP was detected to investigate the binding between Vap7 and the proteins. The black spots represent Vap7 bound to the proteins.

To identify the VEGF-specific aptamers, we performed aptamer blotting assays for all the clones by using 5 pmol of VEGF_165_ immobilized on a membrane. The results showed that Vap7 appeared to bind to the membrane-immobilized VEGF165 (data not shown). We also conducted an aptamer blotting assay to check whether or not Vap7 bound to VEGF_121_ (Figure 1), and we found that Vap7 is capable of binding to both VEGF_121_ and VEGF_165_.

### 2.2. Characterization and Improvement of the Obtained Aptamer

**Figure 2:**
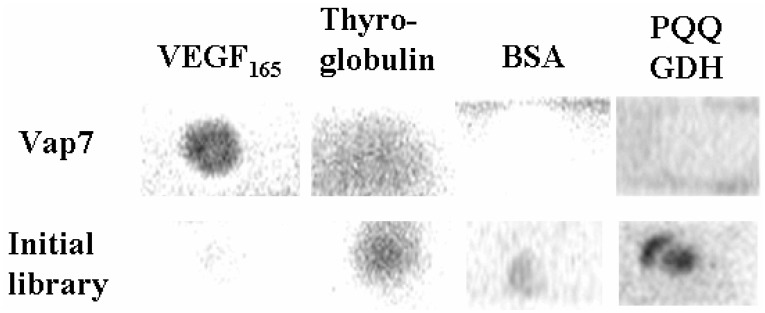
Evaluation of specificity of Vap7. A 5-pmol aliquot of each of the proteins was immobilized on a single membrane. The membrane was incubated sequentially with 100 nM of the nucleic acid modified with FITC and with HRP-labeled anti-FITC antibody. Finally, the chemiluminescence generated by HRP was detected to identify binding between the nucleic acids and the proteins.

To investigate the specificity of Vap7, we performed a competitive aptamer blotting assay by using thyroglobulin, bovine serum albumin (BSA), and pyrroloquinoline quinone glucose dehydrogenase (PQQGDH) as competitors. Thyroglobulin is a marker protein that is used for cancer diagnosis, BSA is a protein that is used as a blocking protein in the aptamer blotting assay, and PQQGDH is an enzyme that we used for labeling of aptamers in a previous study [16]. Vap7 did not bind to these competitors, but it bound specifically to VEGF_165_ (Figure 2). These results suggested that Vap7 is specific to VEGF.

Because Vap7 can bind to both VEGF_121_ and VEGF_165_, it may recognize the RBD, which is the common portion of these two proteins. The affinity of an aptamer is closely related to the accessibility of a target molecule to the aptamer, so it is conceivable that we could optimize the structure of an aptamer to improve this accessibility. The deletion of excess sequences that do not contribute to binding between the aptamer and the target molecule should therefore improve the accessibility of the target to the aptamer. We therefore investigated the structure of Vap7 to identify those sequences that are important for the affinity of this aptamer.

**Figure 3:**
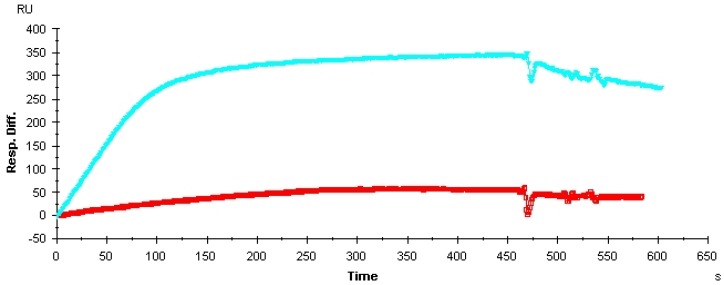
Change in the SPR spectrum of Vap7 depending to presence of KCl. VEGF_165_ (10 pmol) was immobilized on a sensor chip according to the manufacturer’s protocol. Vap7 was diluted to 10 µM with TBSE or TBSE–KCl and then folded by heating. The analyte used for SPR was diluted to 500 nM with the same buffer that was used for folding.The light blue line is the spectrum of Vap7 folded in TBSE containing 50 mM of KCl, and the red line is that of Vap7 folded in TBSE alone.

The M-fold program [17] predicted that Vap7 should have two secondary structures that each contain a stem–loop structure (Supplementary Data 1). QGRS Mapper [18], on the other hand, suggested that Vap7 folds into G-quadruplex structures (Supplementary Data 2). To determine which of these structures is responsible for the affinity of Vap7 for its target, we tested the affinity of Vap7 folded in the presence or absence of K^+^ by means of surface plasmon resonance (SPR) measurements. We found that Vap7 folded in Tris-buffered saline containing EDTA (TBSE) bound to VEGF_165_ less tightly than Vap7 folded in TBSE containing additional KCl (Figure 3). We therefore assumed that K^+^ is important for the structure of Vap7 when it binds to VEGF. The K^+^cation has been reported to stabilize the G-quadruplex structures of nucleic acids [19]. The experimental results, therefore, suggest that Vap7 recognizes VEGF_165_ by means of G-quadruplex structures. We expected that the affinity of Vap7 should be improved if the G-quadruplex structures were stabilized.

**Figure 4:**
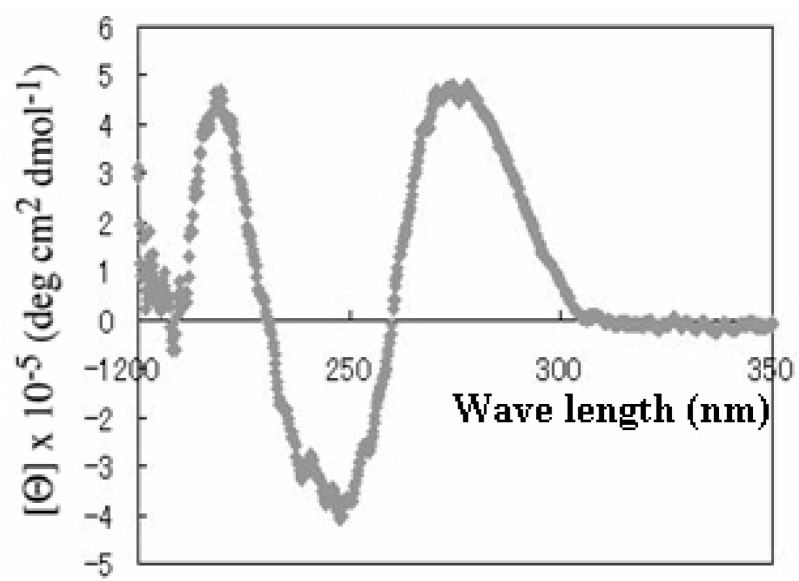
CD spectrum of Vap7. Aptamer Vap7 was diluted to 10 µM with TBSE–KCl and then folded by heating before its CD spectrum was recorded.

Because the results described above suggested that Vap7 might fold into G-quadruplex structures, we examined its circular dichroism (CD) to determine its structure. It is well known that all DNAs that fold into a G-quadruplex structure have a typical CD spectrum [19]. The CD spectrum of Vap7 is shown in Figure 4. There is a negative band at 240 nm and two positive bands at 220 and 270 nm. This spectral feature is similar to that of a parallel G-quadruplex structure previously reported [19]. On the basis of the CD study and the predictions of QGRS Mapper, we designed a truncated mutant (V7t1) of Vap7 that includes all the sequences necessary to generate the presumed G-quadruplex structure (Table 2).

**Table 2:** The sequences of Vap7 andV7t1. The small letters in the sequence of Vap7 shows sub-sequences originating from the primers. Guanine bases that participate in the formation of the G-quadruplex structure are shown in bold face. The underlined sequence in the Vap7 sequence was also found in V7t1.

Name	Sequence (5' to 3')
Vap7	ataccagtctattcaattGCACTCTGTG**GG**GGT**GG**ACG**GG**CCG**GG**Tagatagtatgtgcaatca
V7t1	TGTGGGGGTGGACGGGCCGGGTAGA

**Figure 5:**
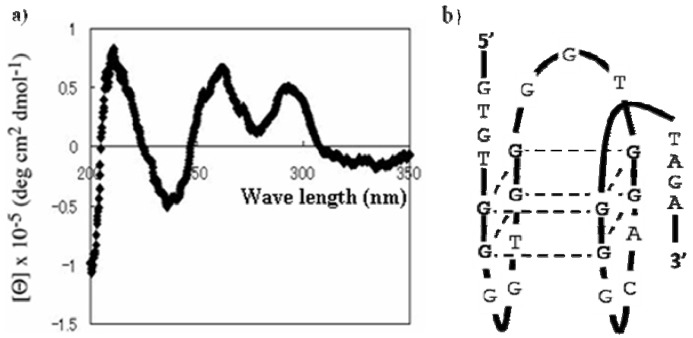
(a) CD spectrum of V7t1. (b) Presumed structure of V7t1. Aptamer V7t1 was diluted to 10 µM with TBSE–KCl and then folded by heating before its CD spectrum was recorded.

Binding between V7t1 and VEGF was checked by means of an aptamer blotting assay (Supplementary Data 3) and SPR measurements (Table 3). The experiments showed that V7t1 binds to both VEGF_121_ and VEGF_165_. Furthermore, the structure of V7t1 was predicted by recording its CD spectrum (Figure 5). Although both Vap7 and V7t1 were capable of binding to VEGF_165_, the CD spectrum of V7t1 differed from that of Vap7.

**Table 3:** Dissociation constants of the aptamers against the VEGF families. The dissociation constants (*K*_D_) were calculated by using the data sets obtained from the SPR measurements.

Aptamer	Target	*K*_D _value
Vap7	VEGF_121_	1.0 nM
Vap7	VEGF_165_	20 nM
V7t1	VEGF_121_	1.1 nM
V7t1	VEGF_165_	1.4 nM

It was surprising that the affinities of the aptamers remained intact after modification of their native structures. Because the G-quadruplex structure of an aptamer is easily generated [20], it is possible that the structure of the aptamer in the binding state is different from that in the nonbinding state. Because V7t1 appears to have sufficient affinity for VEGF_165_, we used V7t1 for specific kinetic investigations.

SPR measurements were carried out to investigate Vap7 and V7t1 in detail (Table 3), and the binding dissociation constant (*K*_D_) for the interaction of VEGF with each of the aptamers was quantified. Vap7 and V7t1 bound to VEGF_165_ with *K*_D_ values of 20 nM and 1.4 nM, respectively, and to VEGF_121_ with *K*_D_ values of 1.0 nM and 1.1 nM, respectively. 

In the aptamer blotting assay, the ability of Vap7 to bind to VEGF_121_ was weaker than that for VEGF_165_ (Figure 1). This finding disagreed with the results of the SPR measurement (Table 3). We believe that this inconsistency is a result of differences in the method used to immobilize the protein. In aptamer blotting, the target protein is immobilized by absorption on the membrane, whereas in SPR measurements, the protein is immobilized on the base through the formation of covalent bonds. This difference in the mechanism of immobilization may have affected the structure of VEGF, thereby causing the difference in the results obtained by aptamer blotting and by SPR measurement.

**Figure 6:**
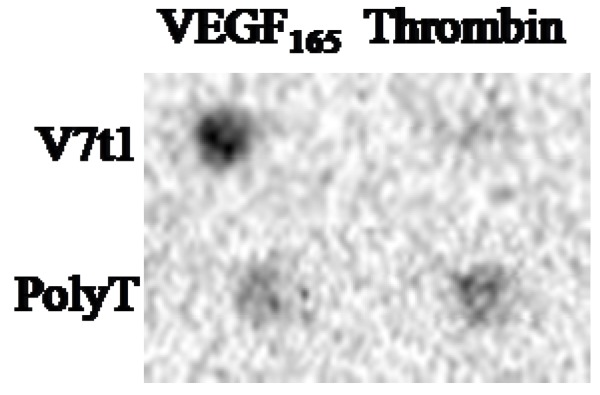
Evaluation of specificity of V7t1. A 5-pmol aliquot of each of proteins was immobilized on a single membrane. The membrane was incubated sequentially with 100 nM of the nucleic acidmodified with FITC and with HRP-labeled anti-FITC antibody. Finally, the chemiluminescence generated by the HRP was detected to identify binding between the nucleic acids and the proteins.

Thrombin is a coagulation protein found in the blood stream that has many roles in the coagulation cascade. It is well known that an anti-thrombin aptamer also forms a G-quadruplex structure. Therefore, to evaluate the specificity of V7t1, we performed an aptamer blotting assay with VEGF_165_ and thrombin (Figure 6). Five pmol each of VEGF_165_ and thrombin were immobilized on the same nitrocellulose membrane. Other parts of assay protocols were as described in the Methods section below.

Because V7t1 appeared to bind to both VEGF_121_ and VEGF_165_ at the RBD, we designed an aptamer heterodimer that contained the previously obtained HBD-binding aptamer (del5-1) [15] and V7t1. The dissociation constant of the aptamer heterodimer against VEGF_165_ was determined by SPR measurements. According to the SPR measurements, the aptamer heterodimer bound to VEGF_165_ with a *K*_D_ value of 4.7 × 10^2^ pM. As a result of dimerization, the *K*_D_ values were improved from 1.4 nM (V7t1) and 3.0 × 10^2^ nM (del5-1) to 4.7 × 10^2^ pM. The two aptamers were linked through ten thymine base units. It is possible that the affinity could be further improved by optimizing the length of the unit linking the two aptamers.

## 3. Experimental

### 3.1. Materials

All the synthetic oligonucleotides were purchased from Invitrogen Japan (Tokyo, Japan). Recombinant human VEGF_165_ (expressed from Sf21 insect cells) and VEGF_121_ (expressed from *Escherichia coli*) were purchased from R&D Systems (Minneapolis, MN, USA) as carrier-free lyophilized powders. These proteins were resuspended in TBSE (10 mM Tris/HCl, 100 mM NaCl, and 0.05 mM EDTA; pH 7.0).

### 3.2. Methods

#### 3.2.1. SELEX Protocol

An FITC-labeled single-strand DNA library containing a 30-mer randomized region and an 18-mer primer-binding region at the two ends, respectively, was used as the first screening library. This library has 1.8 × 10^18^ kinds of sequence variation. The sequence of Fw primer is FITC-ATACCAGTCTATTCAATT from 5' to 3'. And the sequence of Rev primer is Biotin-TGATTGCACATACTATCT from 5' to 3'. That is, the entire sequence of the first screening library is FITC- ATACCAGTCTATTCAATT-N30-AGATAGTATGTGCAATCA aiming at the direction of 5' to 3'. The library was dissolved in TBSE. 

To fold the aptamer, we heated the library at 95 °C for 3 min, and then gradually cooled it to 25 °C at a rate of 2 °C per min. Five pmol of VEGF_121_ were immobilized on a nitrocellulose membrane. Nitrocellulose has a cation on the nitrogen and an anion on the oxygen. Therefore, proteins would be immobilized easily to this membrane by absorption. Next, the membrane was blocked with 10 % (v/v) human serum in TBSTE (TBSE containing 0.05% (v/v) of Tween-20). The folded DNA library and the VEGF_121_ immobilized on the nitrocellulose membrane were incubated together for 1 h at 24°C. After incubation, the membrane was washed twice with TBSTE. Then, the DNAs which were bound to VEGF_121_ were extracted with phenol and precipitated with ethanol. The collected DNAs were amplified with 40 cycles of PCR using Fw and Rev primers and Amplitaq Gold (Applied Biosystems). Then, amplified dsDNA were incubated with Pierce^®^ Avidin Agarose Resins (Thermo) for 1 h at 24 °C. After making avidin-biotin conjugate, dsDNA were separated to ssDNA by treating 0.15 M of NaOH. Separated ssDNA were recovered from the solution and purified with ethanol precipitation. We used the purified ssDNAs as the next screening library. 

The affinities of the DNA libraries for VEGF_121_ were evaluated at each round by aptamer blotting assay [21]. In this evaluation method, after incubation with the DNA library and washing, the membrane was incubated with 0.1% (v/v) HRP-labeled anti-FITC antibody (Dako Cytomation) for 1 h at 24 °C. Then, the membrane was washed with TBSTE twice, and the spots indicating the DNAs bound to VEGF_121_ were visualized with Immobilon Western chemiluminescent HRP substrate (Millipore). 

#### 3.2.2. Aptamer Blotting Assay

The FITC-labeled aptamers were dissolved in TBSE, heated at 95 °C for 3 min, and then gradually cooled, as described above. VEGF_165_ or VEGF_121_ as the target protein was immobilized together with competitor proteins on a nitrocellulose membrane. The membranes were then blocked with 100 ng/mL of BSA in TBSTE, and the aptamers were incubated with the proteins in TBSTE. Next, the membrane was incubated with 0.1% (v/v) HRP-labeled anti-FITC antibody for 1 h at 24 °C then washed twice with TBSTE. The spots corresponding to DNA strands bound to proteins were visualized with Immobilon Western chemiluminescent HRP substrate.

#### 3.2.3. Circular Dichroism Spectroscopy Measurements

The structures of the aptamers were analyzed by circular dichroism (CD) spectroscopy using a JASCO (J-725) spectropolarimeter. To fold the aptamers, they were dissolved in TBSE, heated at95 °C for 3 min, and then gradually cooled as described above. The CD spectra of the folded aptamer samples were recorded at a fixed DNA concentration of 10 µM.

#### 3.2.4. Measurement of the Binding Affinities by Means of Surface Plasmon Resonance

The binding affinities of the aptamers for VEGF were analyzed at 24 °C on a Biacore X instrument (Biacore AB, Sweden). CM5 sensor chips (Biacore AB) were used for all measurements of kinetics, and TBSE was used as the running buffer. Before immobilization of VEGF, the sensor chips were preconditioned with 1 mL of running buffer at a flow rate of 100 μL/min. Next, VEGF (400 U) dissolved into 10 mM acetate buffer (pH 6.0) was immobilized on the sensor chip as stipulated in the manufacturer’s manual. Aptamers at various concentrations were then injected as analytes. A 100-μL aliquot of each of the analytes was added at a flow rate of 20 μL/min for 5 minutes. The association and disassociation of the aptamer–VEGF conjugates were observed for 5 minutes each. The surface was regenerated by treating it with 5 μL of regeneration buffer (5 mM NaOH, 100 mM NaCl) at a flow rate of 20 μL/min. The *K*_D_ values were calculated on the basis of a 1:1 binding model by fitting the association and dissociation rates in the Biacore T100 evaluation software.

## 4. Conclusions

In this study, we aimed to obtain a new RBD-binding aptamer. We have previously succeeded in obtaining an HBD-binding aptamer, and have reported a novel and sensitive method for detecting VEGF_165_ [12]. Our intention was to improve the sensitivity in the detection of VEGF_165_. Because an aptamer dimer that can bind to both the HBD and the RBD will bind to its target across a bigger region of the molecule, its affinity should be higher than that of an aptamer that binds exclusively to HBD or to RBD. To obtain such a dimer, we needed a new aptamer that binds to the RBD in VEGF. We therefore screened aptamers for binding to VEGF_121_, an isoform of VEGF that contains an RBD exclusively. After performing three rounds of screening, we identified a DNA aptamer (Vap7) that bound to both VEGF_121_ and VEGF_165_, and we showed that it bound at the RBD in both isoforms.

To optimize Vap7 for VEGF sensing, we attempted to improve its affinity for VEGF. The affinity of the aptamer should be related to the accessibility of the target molecule. We hypothesized that minimizing the structure of the aptamer to improve the accessibility of its VEGF binding domain should improve its affinity. We predicted the structure of Vap7 to be that of a G-quadruplex by using QGRS Mapper, and we confirmed this prediction by means of CD spectroscopy. We then minimized the sequence of the aptamer with a view to enhancing its affinity for VEGF. When the aptamer structure was suitably modified, the modified aptamer (V7t1) retained its affinity for the VEGF family. Moreover, its affinity for VEGF_165_ was improved from 20 nM to 1.4 nM. We assumed that the induced fit occurred when the obtained aptamers recognized VEGF. 

In this study, tightly binding aptamers were obtained that are expected to be useful for biosensing applications. This is the first report of a VEGF-binding aptamer that folds into a G-quadruplex structure. V7t1, the truncated mutant of Vap7, has a short sequence (29-mer) and a strong affinity for VEGF (*K*_D_ = 1.1 nM for VEGF_1__21_ and *K*_D_ = 1.4 nM for VEGF_165_). An aptamer heterodimer was also constructed, and this showed an even higher binding ability for VEGF_165_ (*K*_D_ = 4.7 × 10^2^ pM), as we had expected.

## Supplementary Materials

Supplementary date can be see downloaded at http://www.mdpi.com/1420-3049/15/1/215/s1/.
