# Supplementary material for: Screening and Improvement of an Anti-VEGF DNA Aptamer
Source: Molecules. 2010 Jan 7;15(1):215–25. doi: 10.3390/molecules15010215 (PMC6256979; doi:10.3390/molecules15010215)

Supplementary data 1. Predicted secondary structure of Vap7

M-fold was used as prediction program. And data sets of folding condition noted below.
Folding Vap7 at 25 °C. [Na+] = 100 mM, [Mg++] = 0.0 M

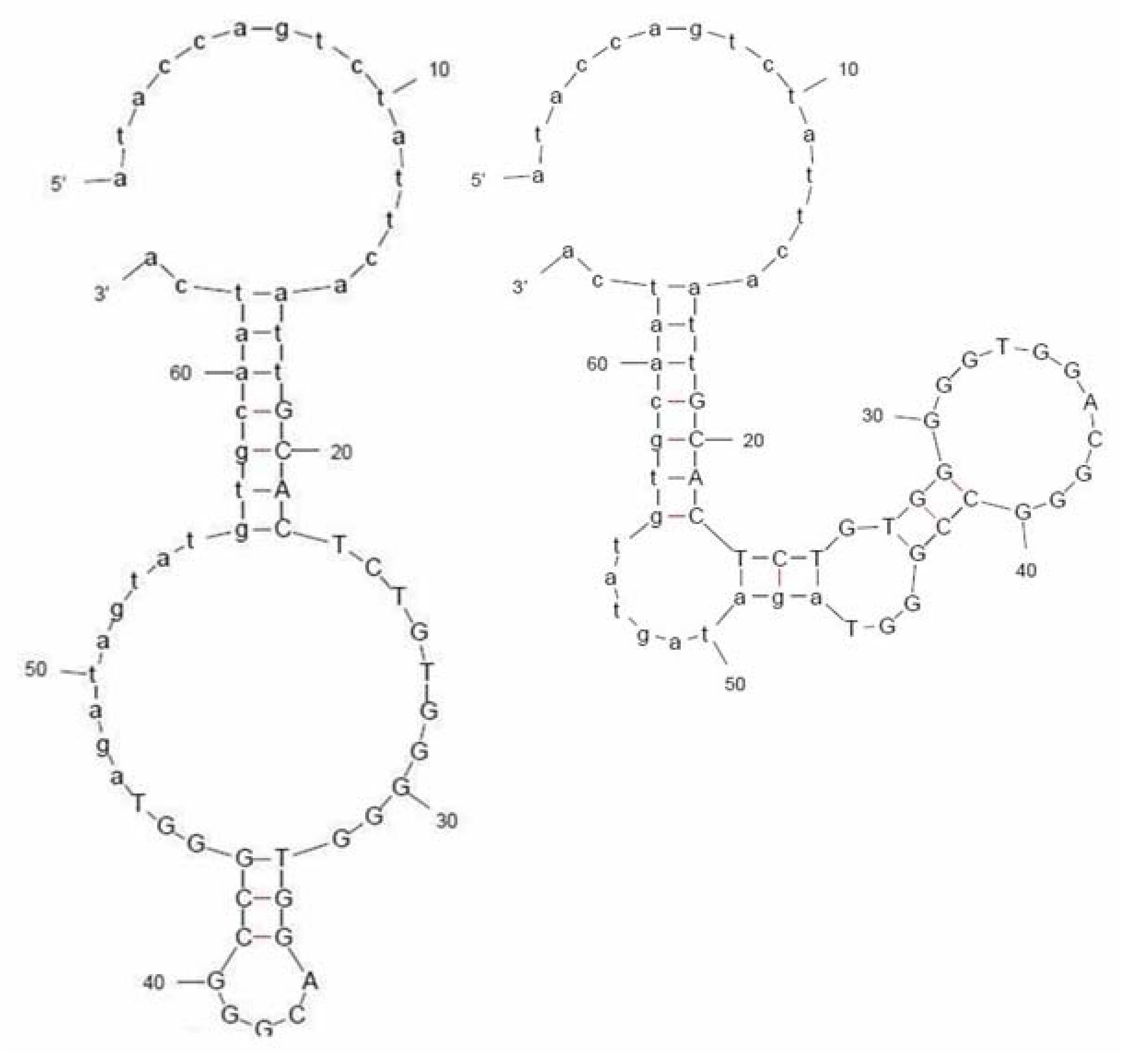

Supplemental data 2. Result of structural prediction by QGRS mapper

Underlined G characters are predicted to form G-quadruplex structure. G-score is meaning the tendency of G-quadruplex structure constructing.

Data View

Search Parameters: QGRS Max Length: 30 | Min G-Group Size: 2 | Loop size: from 0 to 36 | Loop search string:

QGRS sequences found (overlaps included)

Position	Length	QGRS	G-Score
29	17	**GGGGTGGACGGGCCGGG**	21
28	12	GGGGGTGGACGG	20
28	18	GGGGGTGGACGGGCCGGG	20
29	16	GGGGTGGACGGGCCGG	20
29	16	GGGGTGGACGGGCCGG	20
30	15	GGGTGGACGGGCCGG	20
30	15	GGGTGGACGGGCCGG	20
30	16	GGGTGGACGGGCCGGG	20
28	12	GGGGGTGGACGG	19
28	13	GGGGGTGGACGGG	19
28	17	GGGGGTGGACGGGCCGG	19
28	17	GGGGGTGGACGGGCCGG	19
28	18	GGGGGTGGACGGGCCGGG	19
29	11	GGGGTGGACGG	19
29	17	GGGGTGGACGGGCCGGG	19
30	16	GGGTGGACGGGCCGGG	19
31	14	GGTGGACGGGCCGG	19
31	14	GGTGGACGGGCCGG	19
31	15	GGTGGACGGGCCGGG	19
28	13	GGGGGTGGACGGG	18

29	12	GGGGTGGACGGG	18
31	15	GGTGGACGGGCCGGG	18
28	17	GGGGGTGGACGGGCCGG	17
28	18	GGGGGTGGACGGGCCGGG	17
28	17	GGGGGTGGACGGGCCGG	16
28	18	GGGGGTGGACGGGCCGGG	16
29	16	GGGGTGGACGGGCCGG	16
29	17	GGGGTGGACGGGCCGGG	16
28	17	GGGGGTGGACGGGCCGG	15
28	17	GGGGGTGGACGGGCCGG	15
28	18	GGGGGTGGACGGGCCGGG	15
29	16	GGGGTGGACGGGCCGG	15
29	17	GGGGTGGACGGGCCGGG	15
28	17	GGGGGTGGACGGGCCGG	14
28	17	GGGGGTGGACGGGCCGG	14
28	18	GGGGGTGGACGGGCCGGG	14
28	18	GGGGGTGGACGGGCCGGG	14
29	16	GGGGTGGACGGGCCGG	14
28	18	GGGGGTGGACGGGCCGGG	13
29	17	GGGGTGGACGGGCCGGG	13

Supplemental data 3. Result of binding confirmation between V7t1 and VEGF

20 pmol of $VEGF_{121}$, 5 pmol of $VEGF_{165}$ and 20 pmol of PQQGDH were immobilized on nitrocellulose membrane. Then 500 nM of V7t1 were incubated with these membranes. Finally we detected chemiluminescence whether V7t1 binds to proteins or not. Black spots represent binding between V7t1 and each protein.

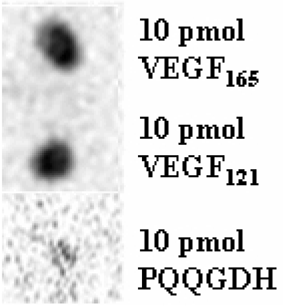

Supplement: Supplementary file 1 [file molecules-15-00215-s001.pdf]
